# Supplementary material for: The ER Unfolded Protein Response Effector, ATF6, Reduces Cardiac Fibrosis and Decreases Activation of Cardiac Fibroblasts
Source: Int J Mol Sci. 2020 Feb 18;21(4):1373. doi: 10.3390/ijms21041373 (PMC7073073; doi:10.3390/ijms21041373)
Supplement: Supplementary file 1 [file ijms-21-01373-s001.pdf]

International Journal of *Molecular Sciences*

MDPI

Supplementary Materials: Supplementary materials can be found at www.mdpi.com/xxx/s1.

Supplement Figure 1

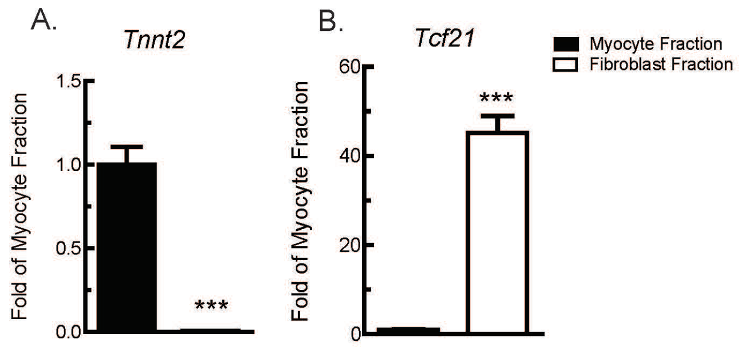

Supplement Figure 1. qRT-PCR of different fractions isolated from a WT adult mouse heart Fractions are separated by gravity sedimentation as described in Methods. The pellet contains the adult myocytes (black bars) and the supernatant contains the non-myocyte fraction. Culturing the non-myocyte fraction for one week (white bars), with regular media changes, allows for the removal of debris and dead myocytes and the enrichment of cardiac fibroblasts (AMVF). (A) Post-isolation, the myocyte fraction is positive for the cardiac myocyte marker *Tnnt2* whereas in the cultured fibroblasts, *Tnnt2* signal is almost completely gone, reflecting the death and removal of any remaining myocytes. (B) Post-isolation the myocyte fraction is negligible for the common fibroblast marker *Tcf21* while it is significantly higher in the cultured fibroblast fraction, reflecting fibroblast enrichment over time. ****$p \leq 0.001$ by one-way ANOVA.*

Supplement Figure 2

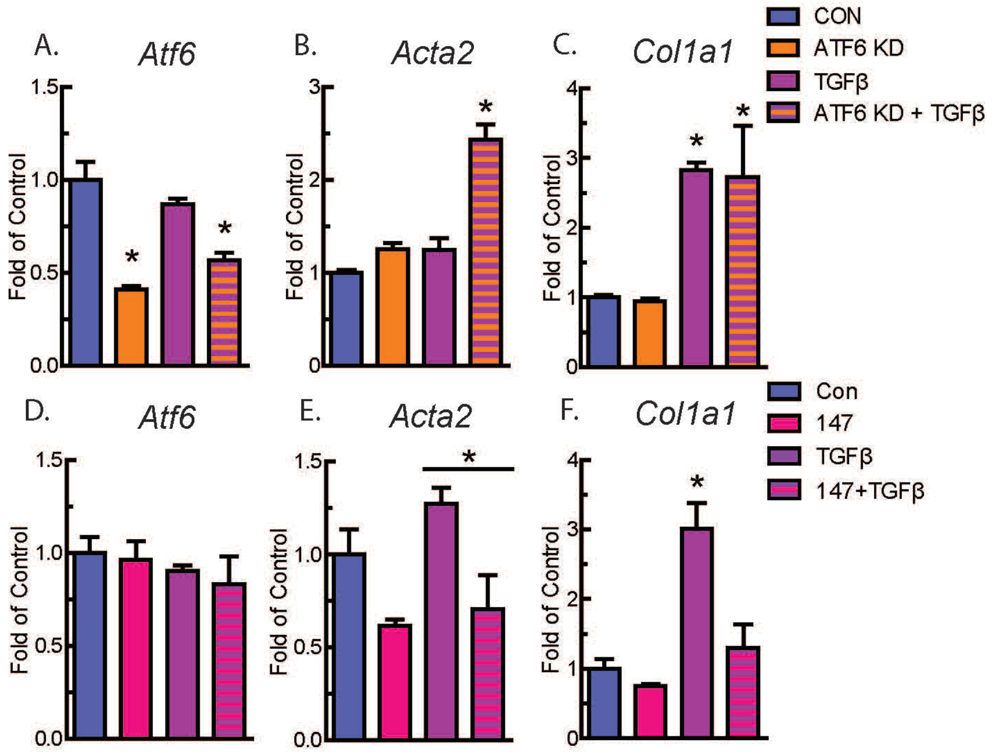

Supplement Figure 2. qRT-PCR of NIH 3T3s with ATF6 gain- or loss-of-function. (A-C) NIH 3T3s were treated ± siRNA targeted to murine ATF6. Control (CON) and siRNA-treated cultures (ATF6 KD) were treated ± 10ng/mL TGFβ for 48 hours, then analyzed by qRT-PCR for *Atf6*, *Acta2*, and *Col1a1*. (G-I) NIH 3T3s were treated ± 10μM compound 147, a pharmacological activator of ATF6. Control (CON) and 147-treated cultures (147) were co-treated ± 10ng/mL TGFβ for 48 hours, then analyzed by qRT-PCR for *Atf6*, *Acta*, and *Col1a1*. *$p \leq 0.05$ by one-way ANOVA.*

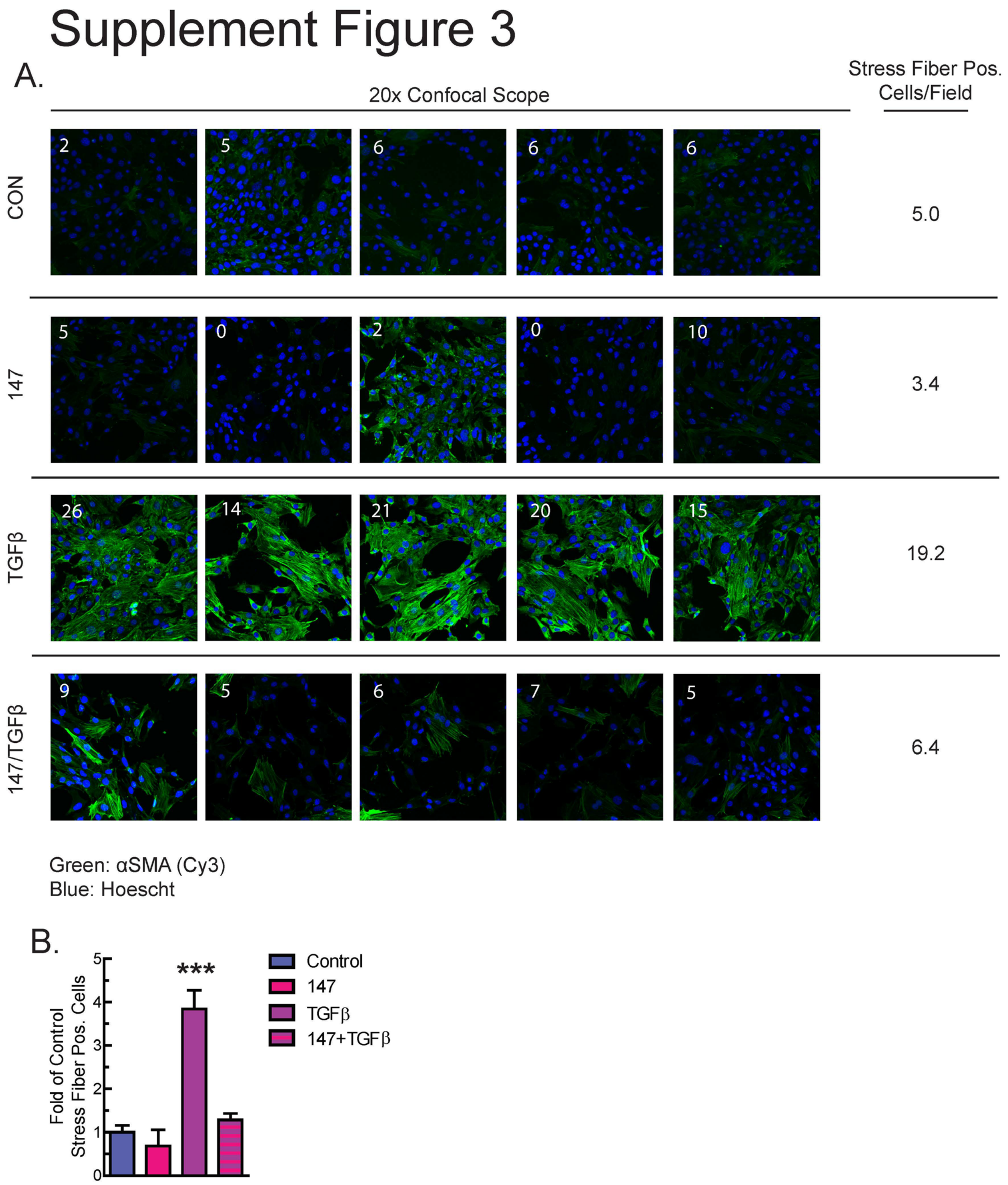

Supplement Figure 3. Effects of activating ATF6 on stress fiber formation in NIH 3T3s (A) NIH 3T3s were treated with ± 10µM compound 147 and ±10ng/mL TGFβ for 48 hours, then analyzed by actin staining for stress fiber formation, which is quantified in (B). All images in (A) were taken with a 20x objective on a confocal scope. In (A) the number in each field represents the number of cells that were stress fiber-positive in that field. The number to the right is the average number of stress-positive cell per field, quantified in (B). *** *$p \leq 0.001$ by one-way ANOVA.*
